# Supplementary material for: TRIM32 inhibits Venezuelan Equine Encephalitis Virus Infection by targeting a late step in viral entry
Source: bioRxiv. 2024 Jun 4:2024.06.04.597282. Preprint. [Version 1] doi: 10.1101/2024.06.04.597282 (PMC11185716; doi:10.1101/2024.06.04.597282)
Supplement: Supplement 1 [file NIHPP2024.06.04.597282v1-supplement-1.pdf]

bioRxiv preprint doi: https://doi.org/10.1101/2024.06.04.597282; this version posted June 4, 2024. The copyright holder for this preprint (which was not certified by peer review) is the author/funder, who has granted bioRxiv a license to display the preprint in perpetuity. It is made available under aCC-BY 4.0 International license.

Supporting information

S1 Fig. Knockout of STING in U_2OS-TRIM32 or U-2OS-Fluc cells. Cells were infected with the indicated viruses and virus infectivity was quantified by flow cytometry.

S2 Fig. Schematic illustration of VEEV-NLuc/Cap and viral protein analysis. A. Illustration of VEEV-NLuc/Cap. B. VEEV-TC83 and VEEV-NLuc/Cap were purified by ultracentrifugation, ponceau S staining. Western blot analysis using antibody against viral capsid protein was employed to analyze viral proteins from virions.

S3 Fig. Proximity labeling proteomics approach to identify TRIM32 interacting proteins. A. Western blot analysis of TRIM32-TurboID system. HeLa-TRIM32.TurboID.HA or HeLa-TurboID.HA cells were mock infected or infected with VEEV-TC83-GFP at MOI of 25 for 6 hrs in the presence of biotin at a concentration of 500μM. The biotinylated proteins were purified by using Streptavidin MagBeads. B. The enriched proteins in each groups were analyzed by using Venny 2.1 (Venny 2.1.0 (csic.es)).

S4 Fig. Schematic illustration of VEEV-nsP3/NLuc and related workflow. SGp: subgenomic promoter, NLuc, Nanoluciferase.

S Table DNA oligos used in this study.

bioRxiv preprint doi: https://doi.org/10.1101/2024.06.04.597282; this version posted June 4, 2024. The copyright holder for this preprint (which was not certified by peer review) is the author/funder, who has granted bioRxiv a license to display the preprint in perpetuity. It is made available under aCC-BY 4.0 International license.

S1 Fig

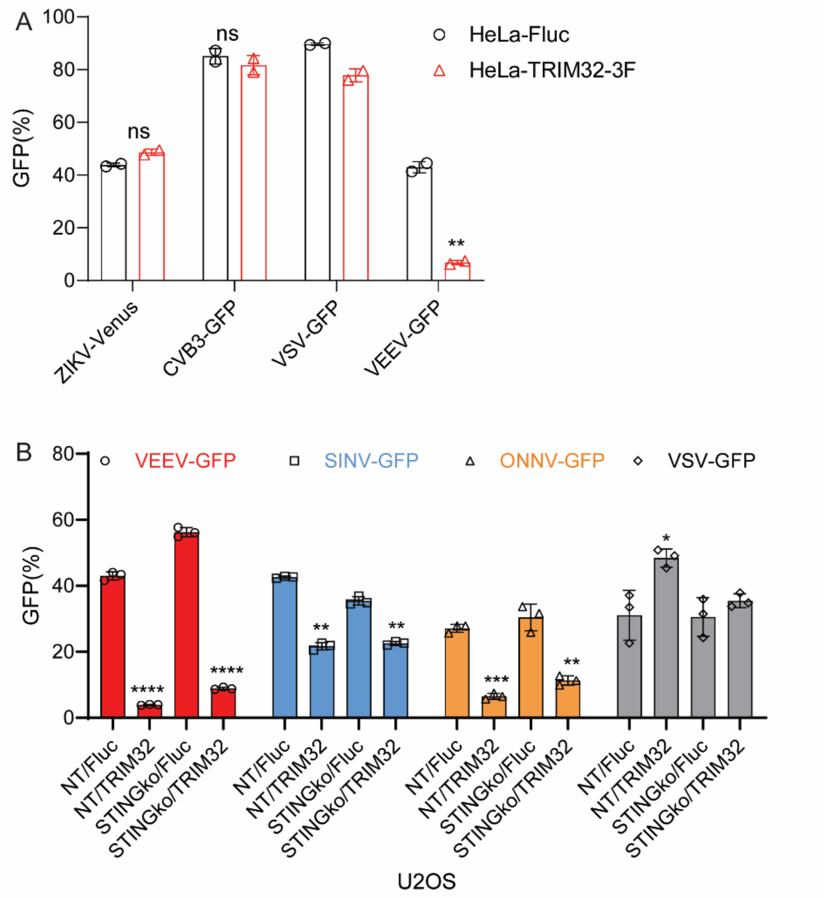

S1 Fig. A. HeLa-Fluc or HeLa-TRIM32-3F were infected with the indicated viruses, and viral infectivity was quantified by flow cytometry. B. STING was silenced in U2-OS-TRIM32 or U2-OS-Fluc cells. Cells were infected with the indicated viruses, and virus infectivity was quantified by flow cytometry. Statistical significance was determined by unpaired students' t-test for A and B (*P<0.05,**P<0.01, ***P<0.001, ****P<0.0001). ns, no significant..

bioRxiv preprint doi: https://doi.org/10.1101/2024.06.04.597282; this version posted June 4, 2024. The copyright holder for this preprint (which was not certified by peer review) is the author/funder, who has granted bioRxiv a license to display the preprint in perpetuity. It is made available under aCC-BY 4.0 International license.

S2 Fig

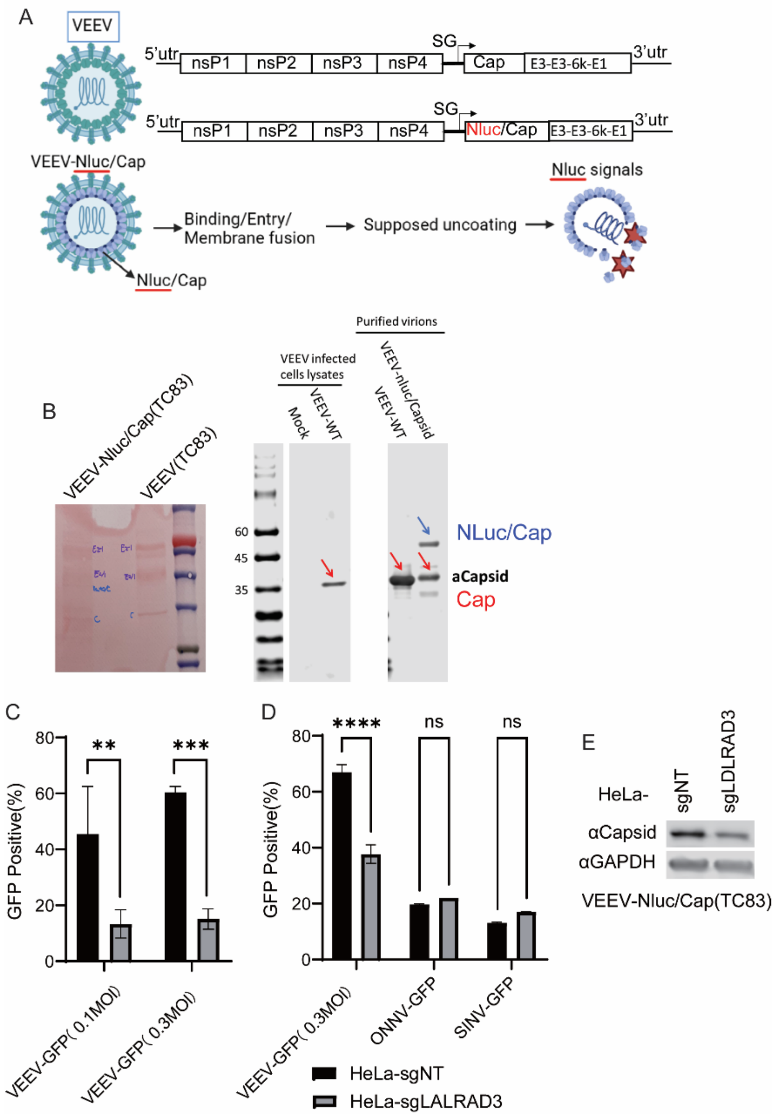

S2 Fig. A. Illustration of VEEV-NLuc/Cap. B. VEEV-TC83 and VEEV-NLuc/Cap was purified by ultracentrifugation, and virion proteins were visualized by ponceau S staining and Western blot analysis using antibody against viral capsid protein. C. HeLa cells stable expressing dual-sgRNA targeting VEEV receptor LDLRAD3 or non-specific targeting sgRNA were infected VEEV-TC83-GFP at the indicated MOI for 24h, and virus infectivity was quantified by flow cytometry. D. HeLa cells stable expressing dual-sgRNA targeting VEEV receptor LDLRAD3 or non-specific targeting sgRNA were infected VEEV-TC83-GFP, SINV-GFP, and ONNV-GFP at MOI of 0.1 for 24h, and virus infectivity was quantified by flow cytometry. Statistical significance was determined by unpaired students' t-test for C and D (**P<0.01, ***P<0.001, ****P<0.0001). ns, no significant.

bioRxiv preprint doi: https://doi.org/10.1101/2024.06.04.597282; this version posted June 4, 2024. The copyright holder for this preprint (which was not certified by peer review) is the author/funder, who has granted bioRxiv a license to display the preprint in perpetuity. It is made available under aCC-BY 4.0 International license.

S3 Fig

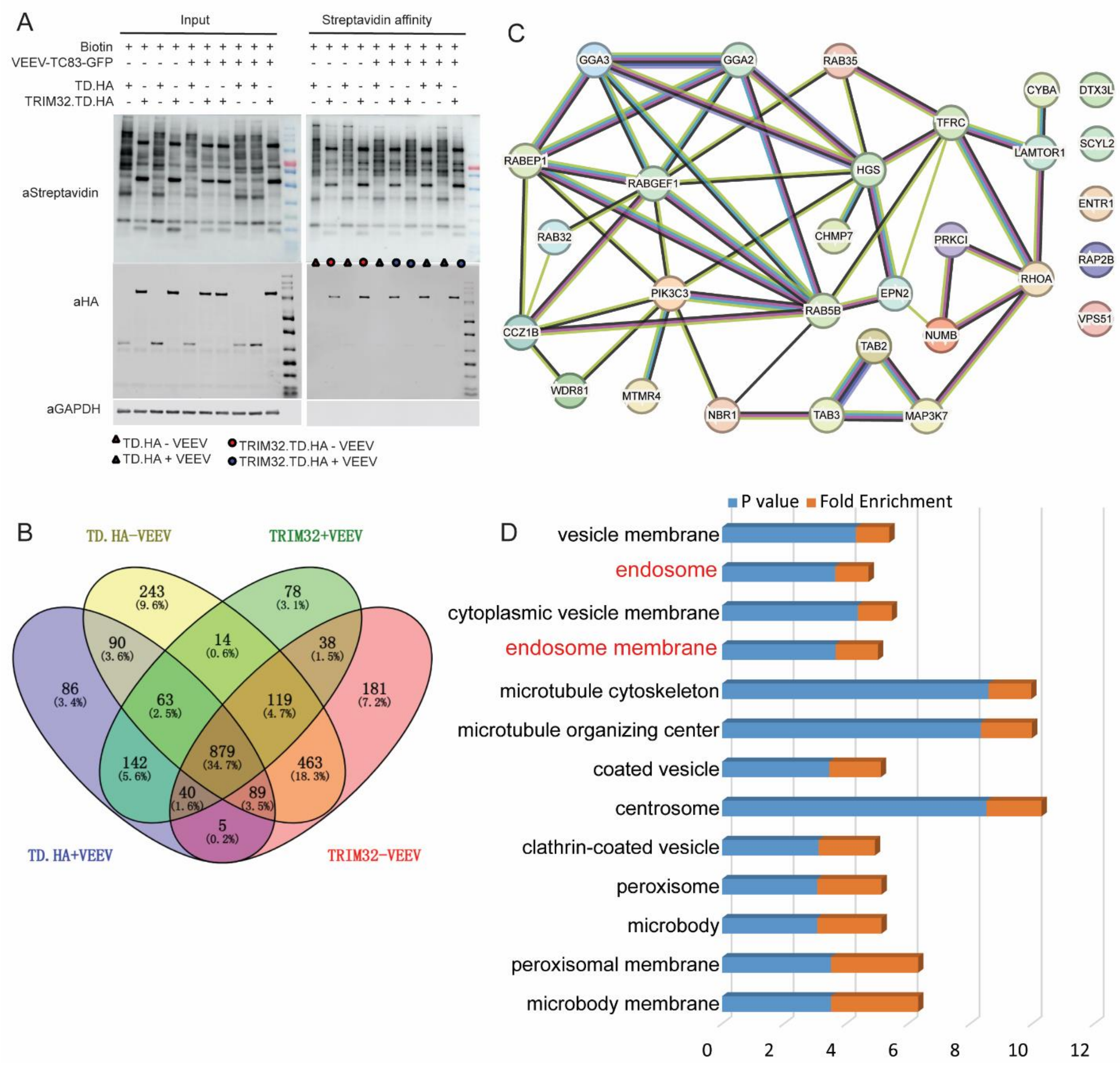

S3 Fig. Proximity labeling proteomics approach to identify TRIM32 interacting proteins. A. Western blot analysis of TRIM32-TurboID system. HeLa-TRIM32.TurboID.HA or HeLa-TurboID.HA cells were infected with or without VEEV-TC83-GFP at MOI of 25 for 6 hrs in the presence of biotin at a concentration of 500μM. The biotinylated proteins were purified by using Streptavidin MagBeads. B. The enriched proteins in each group were analyzed by using Venny 2.1 (Venny 2.1.0 (csic.es) . **C.** The cellular component analysis of TRIM32-enriched proteins by using PANTHER [70]. **D.** The network of TRIM32 proximity labeled endosome associated proteins.

bioRxiv preprint doi: https://doi.org/10.1101/2024.06.04.597282; this version posted June 4, 2024. The copyright holder for this preprint (which was not certified by peer review) is the author/funder, who has granted bioRxiv a license to display the preprint in perpetuity. It is made available under aCC-BY 4.0 International license.

S4 Fig

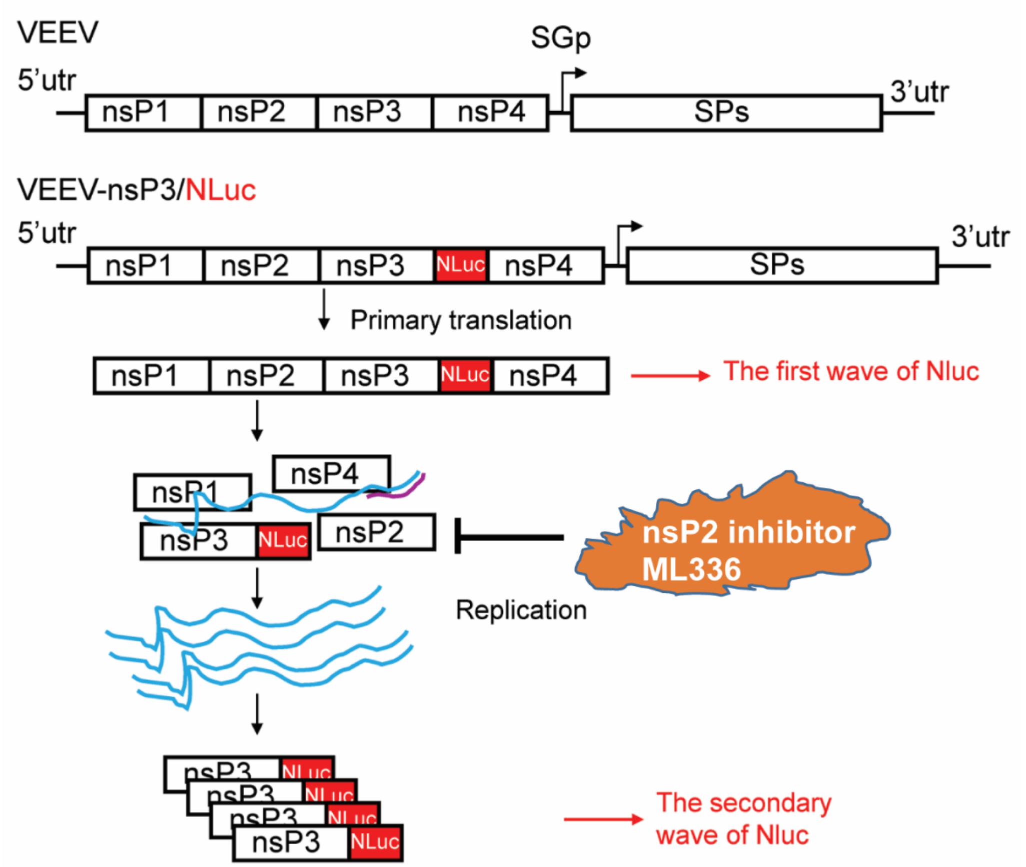

854
855 **S4 Fig.** Schematic of VEEV-nsP3/NLuc and related workflow. SGp: subgenomic promoter, NLuc,
856 Nanoluciferase.
